# Pulmonologists Adherence to the Chronic Obstructive Pulmonary Disease GOLD Guidelines: A Goal to Improve

**DOI:** 10.3390/medicina56090422

**Published:** 2020-08-20

**Authors:** Ruxandra-Mioara Rajnoveanu, Armand-Gabriel Rajnoveanu, Andreea-Bianca Ardelean, Doina Adina Todea, Carmen-Monica Pop, Sabina Antonela Antoniu, Nicoleta Stefania Motoc, Ana Florica Chis, Ariadna Petronela Fildan, Milena Adina Man

**Affiliations:** 1 Pneumology Department, Faculty of Medicine, Iuliu Hatieganu University of Medicine and Pharmacy, 6 BP Hasdeu St, 400371 Cluj-Napoca, Romania; andra_redro@yahoo.com (R.-M.R.); doina_adina@yahoo.com (D.A.T.); cpop@umfcluj.ro (C.-M.P.); motoc_nicoleta@yahoo.com (N.S.M.); anna_f_rebrean@yahoo.com (A.F.C.); manmilena50@yahoo.com (M.A.M.); 2 Occupational Medicine Department, Faculty of Medicine, Iuliu Hatieganu University of Medicine and Pharmacy, 3-5 Clinicilor St, 400006 Cluj-Napoca, Romania; 3 Gastroenterology Department, Faculty of Medicine, Iuliu Hatieganu University of Medicine and Pharmacy, 8 Victor Babes St, 400012 Cluj-Napoca, Romania; andreeabardelean@gmail.com; 4 Nursing/Palliative Care, 2nd Department of Medicine, Gr T Popa University of Medicine and Pharmacy, 16 Universitatii St, 700337 Iasi, Romania; sabina.antoniu@outlook.com; 5 Internal Medicine Department, Faculty of Medicine, Ovidius University, 124 Mamaia Bld, 900527 Constanta, Romania; ariadnapetrofildan@yahoo.com

**Keywords:** adherence, pulmonologist, chronic obstructive pulmonary disease, guidelines

## Abstract

*Background and objectives:* Data about pulmonologist adherence to the Global Initiative for Chronic Obstructive Lung Disease (GOLD) guidelines showed a great variability and cannot be extrapolated. The present study investigates the current pharmacological prescribing practices in the treatment of chronic obstructive pulmonary disease (COPD) according to the 2017 GOLD guidelines, to determine the level of pulmonologist adherence and to identify possible factors that influence physician adherence. *Materials and methods:* This retrospective study took place between 1 February and 30 April 2018 in Pneumophtysiology Clinical Hospital Cluj-Napoca. We included 348 stable COPD outpatients classified according to the 2017 GOLD strategy in the ABCD risk groups. Pulmonologist adherence was defined as appropriate if the recommended pharmacological therapy was the first- or alternative-choice drug according to the guidelines, and inappropriate (overtreatment, undertreatment) if it was not in line with these recommendations. *Results:* The most prescribed treatment was the combination long-acting beta agonist (LABA) + long-acting antimuscarinic agent (LAMA) (34.77%), followed by LAMA + LABA + inhaled corticosteroid (ICS). Overall, pneumologist adherence was 79.02%. The most inappropriate therapies were in Group B (33.57%), followed by 33.33% in Group A. Compared to Groups C and D (analyzed together), Groups A and B had a 4.65 times higher chance (*p* = 0.0000001) of receiving an inappropriate therapy. Patients with cardiovascular comorbidities had a 1.89 times higher risk of receiving an inappropriate therapy (*p* = 0.021). ICS overprescription was the most common type of inappropriateness (17.81%). Groups C and D had a 3.12 times higher chance of being prescribed ICS compared to Groups A and B (*p* = 0.0000004). *Conclusions:* Pulmonologist adherence to the GOLD guidelines is not optimal and needs to be improved. Among the factors that influence the inappropriateness of COPD treatments, cardiovascular comorbidities and low-risk Groups A and B are important. ICS represent the most prescribed overtreatment. Further multicentric studies are needed to evaluate all factors that might influence the adherence rate.

## 1. Introduction

Chronic obstructive pulmonary disease (COPD) remains one of the leading global causes of mortality and morbidity [1], estimated to be the third most common cause of death by 2020 [2]. The 2011 Global Initiative for Chronic Obstructive Lung Disease (GOLD) guidelines were designed to provide healthcare professionals with the most appropriate recommendations for the diagnosis and management of COPD patients. Since then, this guide, and its major (2006, 2011, and 2017), and minor revisions (almost yearly) [3], have become the most important tool accessed in global clinical practices [4]. Of these, the 2017 revision had particular importance because it refined the previous ABCD assessment considering patient symptoms [5,6], combined with the history of exacerbations, excluding spirometry grading and modifying the pharmacological recommendations [1]. Although the COPD guidelines are important for the management of the disease [4], in a number of countries, low levels of adherence to the guideline recommendations were recorded among doctors treating different patient cohorts [4,7,8,9,10,11,12,13,14], often with overtreatment with inhaled corticosteroids (ICS) [13]. According to the World Health Organization (WHO) [15], factors influencing pulmonologist adherence to the guidelines are very complex, related not only to the physician, but also to the patient, disease, and/or social aspects [14,16]. Regardless of barriers, the suboptimal level of adherence has a negative impact on clinical benefits [15], the risk of acute COPD exacerbations [17,18], and treatment success [14]. On the other hand, by improving adherence, the effects are not only clinical and functional [13,19,20], but also come with economic benefits that decrease direct and indirect healthcare costs [21,22].

Most studies that have investigated physician adherence to the GOLD guidelines were conducted at different levels—regional, national [4,7,8,9,10,11,12,13,23], or even local and, single-centered [14]. The results showed a great variability [22,24], and because of this, they cannot be globally extrapolated. They took place among pulmonologists, but also among other specialties involved in COPD treatments [25], evaluating different GOLD reports [13] mainly in 2009–2018, with a higher interest in the 2017 GOLD strategy [4,11,14,23,26]. Hsieh et al. emphasized the importance of better implementing guidelines in clinical practice by optimizing their dissemination [11]. Better communication is also needed between doctors and the organizations responsible for issuing guidelines to identify adherence barriers and to adapt the recommendations to the requirements of daily medical practices [8]. In light of these observations, it was salutary to evaluate the pulmonologist knowledge and application of COPD management guidelines in Romania, where the overall prevalence of this condition was estimated at 8.13% in the adult population [27]. The present study aimed to: (1) investigate the current pharmacological prescribing practices in COPD treatment according to the 2017 GOLD guidelines, (2) determine the level of pulmonologist adherence, and (3) identify possible factors that influence the adherence to therapeutic recommendations.

## 2. Materials and Methods

### 2.1. Study Selection

This retrospective, observational study took place between 1 February and 30 April 2018 in Pneumophtysiology Clinical Hospital Cluj-Napoca. Outpatient electronic medical records were collected. The inclusion criteria were: patients with a documented diagnosis of COPD confirmed by a pulmonologist on the basis of clinical history, a physical examination, and spirometric evaluation; stable forms; aged ≥ 40 years; classified according to 2017 GOLD guidelines in the ABCD risk groups (by symptoms and exacerbation history). The exclusion criteria were: inpatients, younger than 40 years old, an unconfirmed COPD diagnosis, incomplete medical records (lack of GOLD stage/group), and patients presenting acute COPD exacerbations or a concomitant asthma diagnosis. The study was conducted in accordance with the Declaration of Helsinki, and the protocol was approved by the Ethics Committee of Pneumophtysiology Clinical Hospital Cluj-Napoca (approval number 1689/2018, approval date: 26 January 2018).

### 2.2. Data Collection

A reference number was assigned to each patient corresponding to the order in which they were included in the study. The following data were collected: age, race, gender, location of residency (urban or rural), cigarette-smoking history, spirometry classification of airflow-limitation severity GOLD Grades 1–4, GOLD ABCD risk groups, comorbidities (cardiovascular diseases), the presence of a chronic respiratory failure diagnosis in the patient medical records, first consultation or regular follow-up visit, inhaled pharmacological medications prescribed for COPD as a monotherapy or in different combinations of short-acting beta agonists (SABA), long-acting beta agonists (LABA), short-acting antimuscarinic agents (SAMA), long-acting antimuscarinic agents (LAMA), and inhaled corticosteroids (ICS). Comorbid cardiovascular disease consists of arterial hypertension, ischemic heart disease, heart failure, cor pulmonale, arrhythmias, and valvopathies, as they were mentioned in the patients’ medical records.

### 2.3. Adherence to the GOLD Guidelines

Adherence was defined as appropriate if the recommended pharmacological therapy was the first-choice or alternative-choice drug according to the guidelines, and inappropriate if it was not in line with these recommendations [1]. Furthermore, an inappropriate therapy was classified as overtreatment or undertreatment.

An appropriate therapy was assessed according to specific 2017 GOLD algorithms for the treatment of each risk group (Table 1).

The phosphodiesterase 4 (PDE4) inhibitor was not included in our therapy algorithm, as it was not available in Romania. 

### 2.4. Statistical Analysis

The data were collected in 2016 Microsoft Excel software (version 18.2006.1031.0, Microsoft, Redmond, Washington, DC, USA), and transferred to SPSS software (IBM SPSS Statistics 25.0.0.0, Armonk, New York, NY, USA) for the statistical analysis. The quantitative variables were expressed by their median/mean ± standard deviation (SD) and a 95% confidence interval (CI). The qualitative variables were expressed as absolute and relative numbers (percentages). The used statistical tests were the chi-squared test for frequency comparison, the t-test for independent samples to compare the averages of two samples comprising normally distributed variables, and the Mann–Whitney U test to compare the means of two samples comprising abnormally distributed variables. The level for statistical significance was set at *p* < 0.05.

## 3. Results

The study included 348 patients consulted by 15 pulmonologists; 325 patients were excluded due to a lack of a COPD stage/group being documented in their record. The baseline characteristics of the COPD patients are presented in Table 2. The mean age of the cohort was 68.61 years with a standard deviation of 10.61 years. The male gender was predominant (72.41%) with a 2.6:1 ratio compared to females. All patients were white (100%); 191 (54.89%) patients lived in urban areas, and 157 (45.11%) in rural settings. Among the 348 patients, smoking-history data were available for 235 (67.53%) patients—23.83% were current smokers, 63.40% were former smokers, and 12.77% patients had never smoked. In 113 cases, no information about smoking history was available. The data about smoking pack-years were recorded in only 117 patients, with calculated mean pack-years of 40 ± 14.86. According to the GOLD spirometry grades, 37.83% of patients were classified as GOLD 2, followed by GOLD 3 (26.72%), GOLD 4 (26.15%), and GOLD 1 (9.20%). When classified into the refined 2017 GOLD ABCD groups, most patients were in Group B (41.09%) and Group D (28.45%), followed by Group C (24.34%) and Group A (6.03%). Most current smokers were in Group A (28.57%) and Group B (17.48%); 41.24% of COPD patients presented cardiovascular comorbidities, and 115 (33.04%) cases had chronic respiratory failure.

The percentages of the different therapeutic regimens were different across the GOLD ABCD groups, as listed in Table 3. Overall, the most prescribed maintenance treatment was the combination of LAMA + LABA (34.77%), followed by triple therapy LAMA + LABA + ICS (in two different inhalers) (24.14%), LAMA (18.97%), and LABA + ICS (17.82%). The percentages may not sum up to 100 because of rounding.

**Figure 1:**
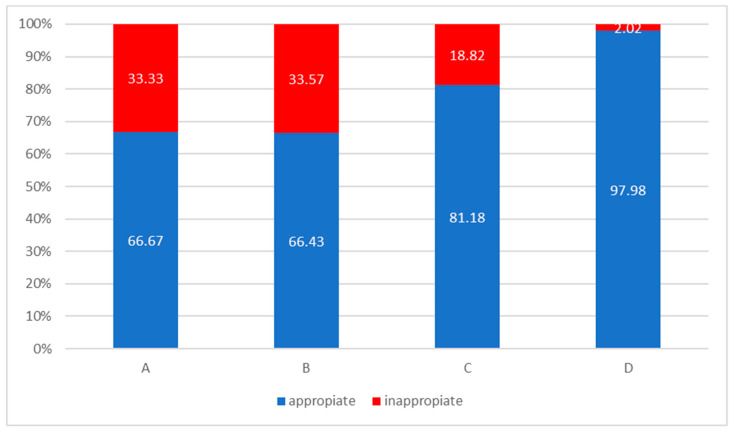
Percentages of appropriate and inappropriate medical therapy according to the 2017 GOLD guidelines (*n* = 348).

From the 348 patients included in the study, 275 patients received the appropriate therapy; therefore, the overall pneumologist adherence to the 2017 GOLD recommendations was 79.02%. The appropriateness and inappropriateness of pharmacological treatments were different for each of the ABCD groups (Figure 1).

The most inappropriate therapy was in Group B patients (33.57%), with overtreatment in 31.46% of cases (0.7% ICS, 20.98% ICS + LABA and 9.79% LAMA + LABA + ICS), and undertreatment in 2.1% of patients. Of Group A patients, 33.33% had inappropriate (overtreatment) prescriptions, with LAMA + LABA (28.57%) and LABA + ICS (4.75%). Of Group C patients, 18.82% were overtreated with triple therapy. Undertreatment was seen in 2.02% of Group D patients. Overall, ICS overprescription was the most common type of inappropriate treatment (17.81%), occurring mostly in Group B patients (31.46%), followed by Group C (18.82%) and Group A patients (4.75%). We applied the chi-squared test to analyze the frequency of ICS prescriptions between the high-risk exacerbation groups (C and D) (analyzed together) in which their prescription was recommended by the GOLD 2017 guidelines compared to the low-risk exacerbation groups (A and B) (analyzed together). The results were statistically significant (*p* = 0.0000004), with patients in Groups C and D having a 3.12 times higher chance of being prescribed ICS compared to Group A and B (odds ratio (OR) 3.12 with a 95% CI, 1.9951–4.8840).

Using statistical tests (a t-test for independent samples, and chi-squared test), we analyzed the variables of 275 patients treated according to the guidelines compared to those with inappropriate prescriptions (Table 4). Group A and B patients (analyzed together) had a significantly statistically higher chance (*p* = 0.0000001) to receive an inappropriate therapy compared to Group C and D patients (analyzed together) with an OR of 4.65 (95% CI, 2.5937–8.3488). The frequency of treatment concordance recorded statistically significant differences between patients with and without cardiovascular comorbidities (*p* = 0.021), with an OR of 1.89 (95% CI, 1.0946–3.27350).

Analysis of the first-choice compared to alternative-choice prescription drug showed statistically significant differences for three variables, as listed in Table 5. Males had a 1.83 times higher chance to receive alternative and not first-choice drugs compared to females (*p* = 0.029), with an OR of 1.83 (95% CI of 1.0568–3.1817). COPD patients with chronic respiratory failure also had a 2.96 times higher chance to receive alternative and not first-choice therapy compared to those without this condition, with an OR of 2.96 (95% CI of 1.6988–5.1455) (*p* = 0.00009). Patients at first consultation had a 2.77 greater chance to receive first-choice therapy and not the alternative variant compared to patients at follow-up visits (*p* = 0.002), with an OR of 2.77 (95% CI of 1.4096–5.4387).

## 4. Discussion

The core findings of our research were the relatively good adherence of pulmonologists to the 2017 GOLD guidelines (79.02%), and the association between inappropriate treatment prescriptions and comorbid cardiovascular disease (1.89 higher risk) on the one hand, and the low-risk exacerbation groups (A and B) (4.65 higher risk) on the other hand. ICS-containing regimens were the most prescribed overtreatment overall (17.81%).

Our cohort of 348 patients had similar demographic and clinical characteristics to those in other studies. The number of our COPD patients was close to that of a North African cohort (296 patients), analyzed by Aissa et al. [14], and more than double the number of patients included in the study of Chinai et al. [26]. The predominance of the male gender (72.4%) was observed in most studies [4,11,13,14,23], while the mean age was similar to the Korean KOCOSS study [4] and the Tunisian study of Aissa [14]. Regarding smoking status, our results were close to those of the Italian study of Palmiotti et al. [13], but the percentage of current smokers (23.83%) was underestimated due to missing data about smoking in about one third (32.37%) of cases. This lack of evidence drew attention to a major deficiency in treating tobacco use and dependence in Romania, where only about 44% of doctors offer smoking-cessation counselling [27,28].

The adherence of pneumologists to the GOLD guidelines represented the primary endpoint of our analysis. According to the definition provided by the United States National Library of Medicine, adherence to medical practice guidelines means “conformity in fulfilling or following official, recognized guidelines, recommendations, protocols” with their application in clinical practice [29]. Though the concept looks clear, it is overly complex [30]. Similar to other studies [4,7,10,11,13,14,23,26], the metric that we used in our study to assess doctor adherence to guidelines was the percentage of pharmacological prescriptions that were in accordance with the recommendations of the GOLD guidelines. In the analysis we did not include factors influencing pulmonologist adherence, a limitation of the study that was also reported by Aissa and colleagues in their research [14]. Overall, pulmonologist adherence to pharmacological therapy, according to the 2017 GOLD guidelines, was 79.02%. This is close to the 70% rate reported by Palmiotti et al. [13], and higher compared to most previously published results, where the adherence level varied between 29.7% in Tunisia and the Maghreb [14], 44.9% in Taiwan [11], 49.6% in South Korea [4], 58.25% in Western Europe and the USA [12], and 61.6% in Turkey [10]. Though there is no standard cut-off for satisfactory adherence [22], our value is improvable. We may assume that the rate of adherence in our study could have a similar explanation to that offered by Palmiotti and colleagues in their study [13]. The study was conducted in a university center where the influx of updated medical information is continuous, with more trainee doctors and important economic resources [13].

The pulmonologist adherence rate was different across the ABCD groups. The highest concordance between the prescribed medication and GOLD 2017 guidelines was seen in high-risk exacerbation patients from Groups D and C (81.18%), followed by prescriptions given to low-risk exacerbation patients from Groups A (66.67%) and B (66.43%). This variation was consistent with that observed in other studies [4,10,12,23], and emphasizes that COPD patients from specific GOLD Groups and in different countries are not appropriately treated by their doctors [14], even with regard to the same therapeutic approach recommended by the GOLD guidelines. Moreover, Group A and B patients (analyzed together) had a 4.65-fold higher chance of receiving inappropriate therapy compared to those from Groups C and D (*p* = 0.0000001). This finding should encourage pulmonologists to more carefully prescribe treatment, not only in high- but also in low-risk groups, knowing that Group B represents the most frequent (41.09%) category of patients [4,11,23]. In contrast to this, most Group D patients received appropriate therapy (97.98%) in line with the GOLD guidelines, a result similar to most related studies [10,13,22,31,32]. No GOLD Group D patient was overtreated, as in the TOLD study [11]. The reasons for all these findings may be the fact that this category of patients, with a high-symptom burden and high-risk of exacerbation, usually receives the maximal therapy (LAMA + LABA + ICS) if further exacerbations occur [1,33], and overtreatment is not possible.

Another significant factor that influenced the appropriateness of the therapy prescription according to the guidelines was the presence of cardiovascular comorbidities. COPD patients frequently present concomitant diseases like cardiovascular diseases [34], lung cancer [35], obstructive sleep apnea [36,37], and depression [38] that may influence the health status, prognosis, and whole course of the disease [1]. In our study, cardiovascular diseases were present in almost half of the COPD patients (42.24%). This comorbidity was a significant factor that influenced pulmonologist adherence to the GOLD guidelines. COPD patients with cardiovascular comorbidities were 1.89 times more likely to be prescribed pharmacological treatments that were inappropriate with the guidelines compared to those without this comorbidity (*p* = 0.02). The predominance of cardiovascular comorbidities was previously observed by Sharif et al. [7], without identifying a statistically significant association between their existence and adherence level. Our findings raise pulmonologists’ awareness to not only identify comorbidities, but to also be more careful when prescribing COPD treatments to patients with concomitant cardiovascular diseases, knowing that treatments for one condition may affect the other; therefore, the appropriate therapy of both conditions is important [34]. Inappropriate treatments in COPD patients with cardiovascular diseases consisted of under- and overtreatments. Undertreatments may be because of historical concern regarding an increased risk of cardiovascular events associated with LAMA and LABA use, particularly at treatment initiation, although the safety profile of this medication administered for a long time seems to be encouraging [34]. In overtreated patients, there may be some concerns regarding the safety of bronchodilators. However, there is conflicting evidence related to the cardiovascular events associated to bronchodilator administration in COPD. Some articles [39,40] suggested an increased risk, while others showed no evidence or even risk reduction [41,42]. Regarding the effect of triple therapy (ICS + LAMA + LABA), the TRIBUTE [43] and FULFIL [44] trials did not confirm excessive cardiovascular risk when compared to LAMA + LABA or ICS + LABA. 

Overall, the most common pharmacological treatment prescribed in our study was the combination of LAMA + LABA (34.77%), similar to the observations of Kim et al. [4]. The motifs are well-documented in the literature, with benefits not only on symptoms, health status, and lung function [1,45], but also on the exacerbation risk when compared to LAMA or LABA + ICS [1,46,47]. Triple therapy (24.14%; ICS + LABA + LAMA) was the second most prescribed regimen in our study. Except for Group D, where it represents an alternative of treatment for some patients [1], it was an inappropriate prescription for Groups B (9.79%) and C (18.82%). These findings were also noted in previous studies [11,12], with the highest frequency (33.94%) recorded by Ding et al. in a multinational study conducted in France, Germany, Italy, Spain, the United Kingdom, and the USA [12]. On a different note, undertreatment (including no therapy) was observed in only 1.44% of our cases, in contrast with the results of Sharif [7] and Aissa [14], where this type of inappropriateness was predominant, and possibly explained by the low rate of COPD diagnosis, especially in the early stages of the disease.

In our study, ICS-containing regimens were prescribed in almost half of the patients (42.24%). Among these prescriptions, overtreatment with ICS (monotherapy or with other drugs, including triple therapy) was seen in 62 (17.81%) patients, which represented the most common type of inappropriateness. The result was not surprising, as it was underlined in most related studies [9,13,48,49,50,51]. The increased prescription frequency of ICS in Groups C and D was expected, but the fact that even patients without history of exacerbations, such as those in Groups A (4.76%) and B (31.46%), received ICS was not in line with the GOLD recommendations. This pattern is not uncommon in real-world practice, as was highlighted in the TOLD study [11,52], the KOCOSS study [4], and in the Portuguese study of Duarte-de-Araujo [23]. The widespread use of ICS in our study could be based on similar explanations in several other papers [13,14,27]: the lack of pulmonologist awareness of the updated GOLD guidelines [53], the long marketing history and information received from pharmaceutical companies [54], the automatic renewal of a prior prescription without an updated assessment [13] or without ICS withdrawal after they were introduced [11,55], pulmonologist preferences [13], the availability of drugs on the market [11], or the belief that ICS is the most beneficial in real life compared to clinical trials [14] and that it should be prescribed regardless of GOLD groups [8,14,56]. Moreover, a look at the local context may indicate other possible reasons why people with a lower GOLD grade are consistently overtreated. A single LABA medication registered on the local market and discontinued availability in drug stores for LABA and LABA + LAMA led to more frequent prescriptions of the ICS + LABA combination. For many years, Romanian pulmonologists treated COPD patients using ICS/LABA medication, and did not update their knowledge on the newer guidelines, which may result in overtreatment with this combination. Without neglecting the well-documented benefits of combining ICS with inhaled bronchodilators on symptoms, the risk of exacerbations and quality of life [4,57], pulmonologists should also weigh the possible side effects like the increased risk of pneumonia or diabetes/poor control of diabetes, oral candidiasis, or mycobacterial infections, including tuberculosis [57], and make the best recommendation to each COPD patient.

Patients at the first consultation had a significantly higher chance to receive first- compared to alternative-choice drugs (*p* = 0.002), a result that seems logical. According to the 2017 GOLD treatment algorithm, both first- and alternative-choice medications are considered appropriate therapies [1]. The male gender and chronic respiratory failure were associated with a higher chance to receive alternative- compared to first-choice drugs. These findings are difficult to interpret because of scarce data in the literature related to these results and the need for further research. Our study, to the best of our knowledge, is the first in Romania to address the problem of pulmonologist adherence to the GOLD 2017 guidelines, which is a strength, along with the inclusion of patients with cardiovascular disease who are systematically excluded in controlled trials. 

Naturally, our study has some limitations. First, this retrospective study was conducted in one center, therefore the results may not be extrapolated. Second, the design of the study, with the inclusion of complete ABCD-categorized patients, may not reflect real life, as COPD patients are still underdiagnosed or have not been classified according to GOLD risk groups. The large number of ineligible patients due to the lack of a COPD stage/group documented in their records may lead to an overestimation of pulmonologist adherence. Another limitation is represented by the lack of patient follow-up, therefore, there is no information about the long-term management of stable COPD is available. Furthermore, the present research does not provide data on the adherence to nonpharmacological treatments or to other pharmacological therapies. Moreover, the study did not assess some important practical issues, like the availability and cost of medications, as the Romanian health-insurance system covers mostly half of the expenses. This can often be a decisive factor in prescribing medication, and may explain pulmonologist deviations from the guideline recommendations.

Lastly, reflecting on our findings, we make some recommendations that may be beneficial for clinical practice in Romania like the need for a more appropriate use of COPD guidelines, starting with a correct and complete diagnosis that leads to an appropriate therapy. Second, particular attention has to be paid by all clinicians to patients in low-risk Groups A and B and to those with concomitant cardiovascular disease.

## 5. Conclusions

Our study outlined that the pulmonologist adherence to the GOLD guidelines is not optimal and needs to be improved. Among factors that influence the inappropriateness of COPD treatment, the presence of cardiovascular comorbidities, and the low-risk groups (A and B) are particularly important. ICS-containing regimens represent the most common overtreatment, even in patients at a low risk of exacerbation. Further multicentric studies are needed to evaluate all factors that might influence adherence rates to reduce the gap between the guideline recommendations and real-life practice.

## Figures and Tables

**Table 1:** Criteria of appropriate and inappropriate treatments according to 2017 Global Initiative for Chronic Obstructive Lung Disease (GOLD) guidelines.

	Guideline-Concordant (Appropriate)		Guideline-Discordant (Inappropriate)	
Group	First Choice	Alternative Choice	Undertreatment	Overtreatment
**A**	SABA or SAMA	Change the bronchodilator	No bronchodilator	LABA + LAMA, ICS-containing regimen
**B**	LAMA or LABA	LAMA + LABA	Only short-acting bronchodilator	ICS + LABA, ICS + LABA + LAMA, ICS + LAMA
**C**	LAMA	LAMA + LABA or ICS/LABA	Only ICS or LABA or SABA	ICS + LABA + LAMA, ICS + LAMA
**D**	LAMA + LABA or LAMA or ICS/LABA	If further exacerbations/symptoms ICS + LABA + LAMA	Only ICS or LABA or SABA, ICS + LAMA	

Abbreviations: GOLD, Global Initiative for Chronic Obstructive Lung Disease; SABA, short-acting beta agonist; LABA, long-acting β2-agonists; LAMA, long-acting muscarinic antagonist; ICS, inhaled corticosteroids; SAMA, short-acting antimuscarinic agents.

**Table 2:** Baseline characteristics of chronic obstructive pulmonary disease (COPD) patients.

Variable	Frequency (*n* = 348)
Age (years, mean (SD))	68.61 (10.61)
Gender	
Male, *n* (%)	252 (72.41)
Female, *n* (%)	96 (27.59)
White race, *n* (%)	348 (100)
Location of residency	
Urban	191 (54.89)
Rural	157 (45.11)
Smoking history, *n* (%)	235 (67.53)
Current	56 (23.83)
Former	149 (63.40)
Never	30 (12.77)
Pack-years (mean (SD)) (*n* = 117)	40 (14.86)
Cardiovascular comorbidity, *n* (%)	147 (42.24)
Chronic respiratory failure, *n* (%)	115 (33.04)
Spirometric GOLD grade, *n* (%)	
GOLD 1	32 (9.20)
GOLD 2	132 (37.93)
GOLD 3	93 (26.72)
GOLD 4	91 (26.15)
GOLD group, *n* (%)	
A	21 (6.03)
B	143 (41.09)
C	85 (24.43)
D	99 (28.45)

SD—standard deviation

**Table 3:** Maintenance pharmacological regimens used for COPD management across the GOLD ABCD groups.

Therapy	All	Group A	Group B	Group C	Group D
SABA, *n* (%)	7 (2.01)	5 (23.81)	1 (0.7)	0 (0)	1 (1.01)
LABA, *n* (%)	4 (1.15)	0 (0)	4 (2.8)	0 (0)	0 (0)
LAMA, *n* (%)	66 (18.97)	9 (42.86)	43 (30.07)	11 (12.94)	3 (3.03)
LAMA + LABA, *n* (%)	121 (34.77)	6 (28.57)	48 (33.57)	41 (48.24)	26 (26.26)
ICS, *n* (%)	1 (0.29)	0 (0)	1 (0.7)	0 (0)	0 (0)
LABA + ICS, *n* (%)	62 (17.82)	1 (4.76)	30 (20.98)	17 (20)	14 (14.14)
LAMA + LABA + ICS, *n* (%)	84 (24.14)	0 (0)	14 (9.79)	16 (18.82)	54 (54.55)
No therapy	3 (0.86)	0 (0)	2 (1.40)	0 (0)	1 (1.01)

**Table 4:** Influencing factors of appropriate or inappropriate therapies according to the 2017 GOLD guidelines.

Variable	Concordant (*n* = 275)	Discordant (*n* = 73)	*p* Value
Age (mean, (SD))	68.90 (10.31)	67.53 (11.66)	0.363 ^a^
Male, *n* (%)	201 (73.09)	51 (69.86)	0.583 ^b^
Rural, *n* (%)	130 (47.27)	27 (36.98)	0.116 ^b^
Chronic respiratory failure, *n* (%)	115 (41.82)	31 (42.47)	0.92 ^b^
Cardiovascular comorbidities, *n* (%)	147 (53.45)	50 (68.49)	0.02 ^b^
First consultation, *n* (%)	41 (14.91)	10 (13.69)	0.794 ^b^
Groups A and B (*n*) vs. Groups C and D (*n*)	109 vs. 166	55 vs. 18	0.0000001 ^b^

^a^ t-test for independent samples; ^b^ chi-squared test.

**Table 5:** Patient characteristics who were prescribed first-choice therapy compared to alternative-choice drugs.

Variable	First-Choice (*n* = 91)	Alternative-Choice (*n* = 184)	*p* Value
Age (mean, (SD))	67.65 (10.82)	69.52 (10.02)	0.171 ^a^
Male, *n* (%)	59 (64.83)	142 (77.17)	0.029 ^b^
Rural, *n* (%)	40 (43.96)	90 (48.91)	0.438 ^b^
Chronic respiratory failure, *n* (%)	23 (25.27)	92 (50)	0.00009 ^b^
Cardiovascular comorbidities, *n* (%)	147 (53.45)	50 (68.49)	0.869 ^b^
First consultation, *n* (%)	41 (14.91)	10 (13.69)	0.002 ^b^

^a^ t-test for independent samples; ^b^ chi-squared test.
